# Acute coronary syndrome and its treatment outcomes in Ethiopia: a systematic review and meta-analysis

**DOI:** 10.1186/s40545-023-00603-7

**Published:** 2023-08-07

**Authors:** Bekalu Kebede, Melese Getachew, Samuel Agegnew, Ephrem Mebratu Dagnew, Dehnnet Abebe, Anteneh Belayneh, Bantayehu Addis Tegegne, Tiringo Kebede, Mekides Kiflu, Yalemgeta Biyazin, Yoseph Merkeb Alamneh

**Affiliations:** 1 Pharmacy Department, Health Science College, Debre Markos University, Debre Markos, Ethiopia; 2 Department of Nursing, Health Science College, Debre Markos University, Debre Markos, Ethiopia; 3 Department of Pediatrics and Child Health Nursing, Health Science College, Debre Markos University, Debre Markos, Ethiopia; 4 Department of Biomedical Sciences, School of Medicine, Debre Markos University, Debre Markos, Ethiopia

**Keywords:** Acute coronary syndrome, Risk factors, Mortality, Management, Meta-analysis, Systematic review, Ethiopia

## Abstract

**Background:**

Acute coronary syndrome (ACS) is the principal cause of death in developing countries including Ethiopia. No study reports the overall patterns of risk factors and burden of in-hospital mortality in Ethiopia. This study, therefore, aimed to assess the magnitude of risk factors, management, and in-hospital mortality of ACS in Ethiopia.

**Methods:**

Electronic searching of articles was conducted using PubMed, Science Direct, EMBASE, Scopus, Hinari, and Google Scholar to access articles conducted in Ethiopia. The Preferred Reporting Items for Systematic Reviews checklist was used for identification, eligibility screening, and selection of articles. Data were extracted with an abstraction form prepared with Microsoft Excel and exported to STATA for analysis. Funnel plot, Begg’s test, and Egger’s test were used to determine publication bias. Heterogeneity between the studies was checked by I^2^ statistic. The pooled prevalence of risk factors and in-hospital mortality of ACS were estimated using a random-effects meta-analysis model.

**Results:**

Most (59.367%) of the patients had ST-segment elevation myocardial infarction (STEMI). Hypertension (54.814%) was the leading risk factor for ACS followed by diabetes mellitus (38.549%). Aspirin (56.903%) and clopidogrel (55.266%) were most frequently used in patients with STEMI ACS, respectively. The pooled proportion of in-hospital mortality of ACS was 14.82% which was higher in patients with STEMI (16.116%).

**Conclusion:**

The rate of in-hospital mortality is still high which was higher in patients with STEMI. Initiation of treatment must consider the heterogeneity of each patient’s risk factor and reperfusion therapy should be implemented in our setting.

**Supplementary Information:**

The online version contains supplementary material available at 10.1186/s40545-023-00603-7.

## Introduction

Acute coronary syndrome (ACS) is a coronary artery disease caused by narrowing or blockage of the coronary artery lumen, resulting in myocardial ischemia or infarction due to insufficient coronary blood perfusion [1, 2]. ACS, including unstable angina (UA), ST-elevation myocardial infarction (STEMI), and non-ST-elevation myocardial infarction (NSTEMI) is the main cause of cardiovascular death and disability in the world [3, 4]. It is associated with an average of 7.4 million deaths worldwide, 21–22% of all deaths in Europe, and 6–10% of death in sub-Saharan Africa [5, 6].

It is an emergency, life-threatening condition, and financial catastrophe due to high out-of-pocket expenditures for in-hospital care [7, 8]. Even though, there is advancement in diagnosis and management of ACS, still it is the most common cause of death around the globe including in Ethiopia [9, 10]. A study done at Addis Cardiac Hospital, Ethiopia showed that among 300 cardiac patients, 162 (53.7%) of them had ACS [11]. Moreover lower-income countries are facing a double burden of disease because of the higher prevalence of both communicable and non-communicable diseases particularly cardiovascular diseases [10]. These result in frequent hospitalizations, high mortality rate, stretching the already limited resources, and are associated with worse treatment outcomes [9, 12].

Even though ACS is a common health problem with devastating consequences, its burden and risk factors are different according to geographical variation [13, 14]. Previous study in sub-Saharan Africa reported that diabetes, hypertension and cigarette smoking still account for the most common predisposing risk factors [6]. Dyslipidemia, obesity, advanced age, family history of ischemic heart disease and sedentary life style are other most common risk factors reported from different studies [2, 7, 10, 13]. More than 90% of risk factors are potentially modifiable risk factors using pharmacological and non-pharmacological methods. However, it is difficult to achieve these outcomes in resource-limited countries because of insufficient laboratory setup, lack of reperfusion therapy, cost of medications, and discontinuity of care [11, 15, 16]. Though several scholars attempted to describe the extent of risk factors, management and in-hospital mortality due to acute coronary syndrome in Ethiopia, there is no a nationwide study on this area; which is an important research gap. Meta-analysis is a key to improving the accuracy of estimates through the use of more data sets. Therefore, this study was aimed to determine the overall magnitude of risk factors, management, and in-hospital mortality of acute coronary syndrome in Ethiopia.

## Materials and methods

### Search strategy

This systematic review and meta-analysis were conducted and reported per the Preferred Reporting Items for Systematic Reviews and Meta-Analysis (PRISMA, 2009) requirements for observational studies [17] (Additional file 1). An electronic search for studies was done by two (BK and MG) of the authors from October 15 to November 30, 2021. PubMed, Science Direct, EMBASE, Cochrane Database, Sci-Hub, Scopus, Africa journal of online library, Hinari, and Google Scholar for free articles were searched. In addition, Addis Ababa, Jimma, and Gondar Universities’ institutional repositories were considered to address unpublished articles in Ethiopia. The searching process was carried out using the full title (“Magnitude of Risk Factors, Management and in Hospital Mortality of Acute coronary syndrome in Ethiopia”) and then keywords (magnitude, risk factors, treatment, mortality, acute coronary syndrome, coronary heart disease, Ethiopia). These different keywords were used individually and in combination using Boolean operators “OR, AND or NOT” as well as medical subject heading [MeSH] terms). In addition, searching reference lists of all the included studies (snowball technique) was done to retrieve other studies that are not addressed by our searching stratagem.

### Study selection criteria

All available studies and data were incorporated based on the following predefined eligibility criteria.

### Inclusion criteria

- *Study setting and period*: All studies conducted in Ethiopia from 2000 to November 30, 2021.
- *Study design*: All facility-based observational studies.
- *Study population*: Age ≥ 18 years old.
- *Article types*: The published and unpublished studies reporting the risk factors of ACS, management, and/or in-hospital mortality.
- *Language*: All searches were limited to articles written in the English language.

### Exclusion criteria

We excluded reviews and systematic review articles, case reports, and case series. In addition, articles available only in abstract form were excluded because it was difficult to evaluate their quality and extract all necessary information. In the case of duplicates, only the most recent or most complete publication for each data set for a specific outcome was selected.

### Outcome variables

The main outcome of this study was the rate of in-hospital mortality. Other secondary outcomes include magnitudes of risk factors and in-hospital management of ACS.

### Data extraction

Essential data were extracted from eligible studies by using Microsoft Excel 2019 spreadsheet format. To ensure data quality and methodological validity, two authors (BK and MG) retrieved the data independently. Data were extracted using the PRISMA standard data extraction format. The following information was considered during data extraction: The last name of the first author and year of publication, the region of the study conducted, study design and period, total sample size, sex, types of ACS, risk factors of ACS, the medication used, overall in-hospital mortality and mortality in each type of ACS. Any discrepancies in the data extraction process were solved through discussion involving all authors.

### Quality assessment tools

Data extraction was accompanied by two authors independently according to the Critical Appraisal Checklist recommended by the Joanna Briggs Institute (JBI) [18]. The JBI checklist was composed of ten questions, the scores ranged from zero to ten. The studies which obtained more than 60% were considered as good quality studies. None of them had poor quality status and all of them were included in the present inquiry. In addition, the Alberta Heritage Foundation for Medical Research (AHFMR) standard quality assessment criteria for primary research papers was used to check the quality of included articles [19]. Accordingly, the quality of articles was stated as high, moderate and, poor quality with respective scores of ≥ 70%, 51–69%, and ≤ 50%.

Moreover, the disagreements were resolved by consensus and decided by taking the average score of the two reviewers.

### Statistical analysis

Data were extracted by using Microsoft excel 2010 format and exported to STATA 14.0 (STATA, College Station, TX, USA) for further analysis. Data were pooled and a random effect meta-analysis with an estimation of DerSimonian and Laird method was applied to determine the pooled prevalence of risk factors, and in-hospital mortality of ACS, and the result was presented using forest plot and odds ratio (OR) with a 95% confident interval (CI), which were used to report the magnitude of the risk factors and mortality in the results of meta-analyses. The pooled prevalence of risk factors and pooled magnitude of in-hospital mortality of ACS were calculated as a weighted average of the individual summary risk factors and a weighted average of the individual summery mortality or ORs), respectively. Similarly, odds ratio of risk factor is simply defined as the ratio of the odds of the risk factor in group (studies) to the odds of the studies without that particular risk factor. Heterogeneity between studies was assessed by computing Chi-square (I^2^) test statistics. The I^2^ values of 0, 25, 50, and 75% were considered as no, low, moderate, and high heterogeneities, respectively [20]; unfortunately, in our study, there was high heterogeneity between the original studies (I^2^ = 95.6%, *p* < 0.001). Subgroup analysis was considered to examine how medications are used and in hospital, mortality varies across different studies based on types of ACS. Begg’s funnel plot and Egger's regression were used to check publication bias and a *p*-value less than < 0.05 was considered statically significant. [21]

## Results

### Characteristics of studies included in the analysis

**Fig. 1:**
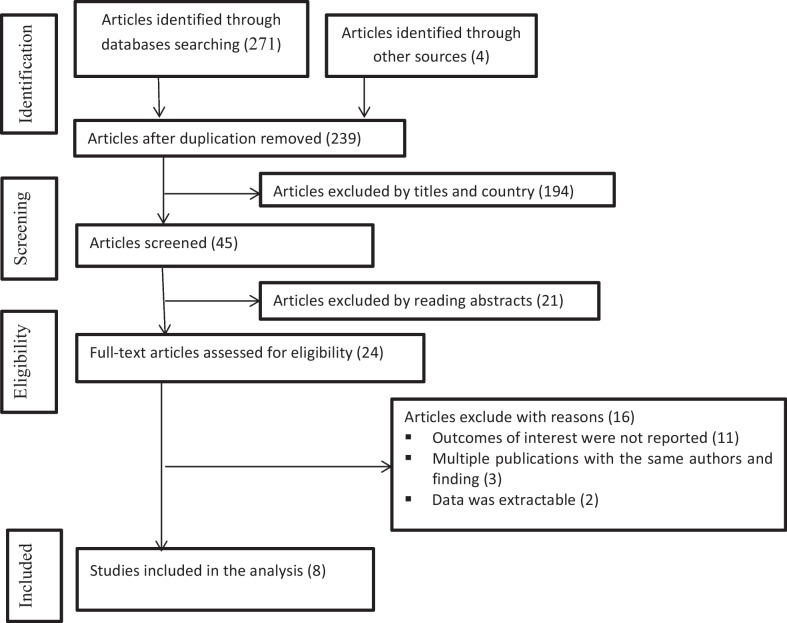
Flow diagram of literature search and study selection based on Preferred Reporting Items for Systematic Review and Meta-Analysis (PRISMA)

**Fig. 2:**
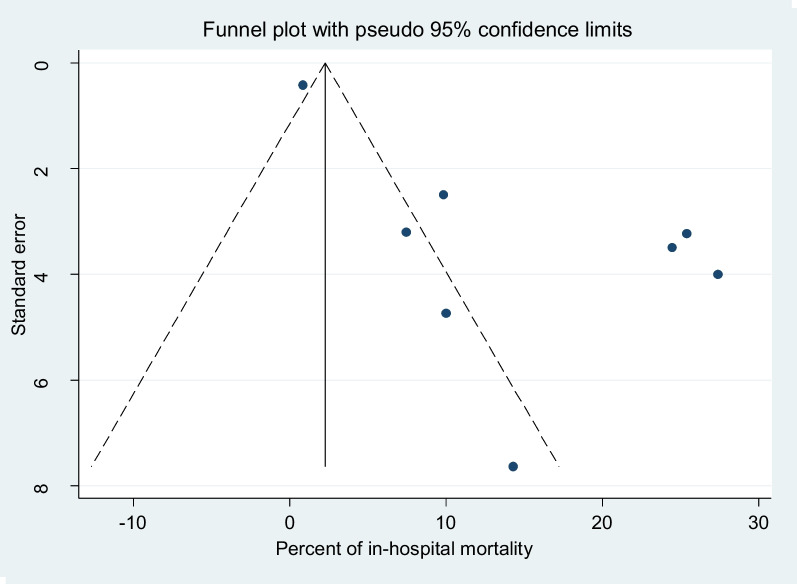
Funnel plot for the publication bias of the included studies for overall all in-hospital mortality

We identified a total of 271 published and 4 from gray literature through database searches. After duplicate papers were removed and a thorough reading of articles’ abstracts, 24 studies were assessed with the eligibility criteria. Accordingly, 16 irrelevant articles were excluded because they did not qualify the eligibility criteria set. Hence, a total of 8 studies were included in the final analysis as they met the inclusion criteria (Fig. 1). The articles were published between 2013 and 2021; however, there is one unpublished article that was obtained from the Addis Ababa University repository. The result of both Begg’s and Egger’s tests showed that there was considerable publication bias at < 0.001 *p*-values and < 0.006, respectively, and assessment of publication bias using a funnel plot in (Fig. 2). Most of the studies (6/8, 75%) were cross-sectional studies, others were longitudinal or retrospective follow-up studies.

More than one-half of the studies were conducted in Addis Ababa city (*n* = 6) others were conducted in the Oromia region (*n* = 1), and Tigray region (*n* = 1). Of a total of 1197 study participants, 813(67.92%) of them were females (Additional file 2). Out of the eight studies included in this review and meta-analyses five studies were single-center [22–26] while the rest three being multi-center. All patients presented with acute coronary syndrome were included in all the eight studies as the number of patients in respective hospitals was limited. Only one study [24] was found to be moderate quality (68.18%) when evaluated with a checklist for assessing the quality of quantitative studies adopted from Kmet and Robert (Additional file 2); whereas, the rest articles had high-quality score.

With regard to diagnostic types of ACS, majority of patients had STEMI (59.367%, 95% CI: 44.558–74.176) followed by NSTEMI (23.365%, 95% CI: 16.559–30.171) and then UA (19.899%, 8.092–31.706) (Table 1).

**Table 1:** Characteristics of studies included in the meta-analysis

Author/s (reference)	Study design	Reginal state	sample size	Male	Female	Prevalence of types of ACS			In-hospital mortality			
						STEMI	NSTEMI	UA	Total	STEMI	NSTEMI	UA
Desta et al., 2020 (21)	R. cross-sectional study	Tigray	151	109.00	42.00	72.80%	15.20%	12.0%	37.00	25.00	9.00	3.00
Bogale et al., 2019 (22)	R. cross-sectional	AA	124	94.00	30.00	72.60%	16.10%	11.30%	34.00	32.00	1.00	1.00
Tsegaye et al., 2021 (23)	R. cohort study	AA	471	315.00	156.00	32.98%	28.30%	38.9%	4.00	N/A	N/A	N/A
Hailu et al., *2020* (24)	R. cross-sectional	AA	67	151	29.00	N/A	N/A	N/A	5.00	N/A	N/A	N/A
Fanta et al., 2021 (25)	P. observational	Oromia	181	113.00	68.00	61%	39%	0	46.00	35.00	11.00	0.00
Giday et al., 2013 (26)	R. cross-sectional	AA	21	16	5.00	62%	28.60%	9.40%	3.00	2.00	1.00	0.00
Wakwaya et al., 2019 (27)	R. cross-sectional	AA	40	29.00	11.00	67.50%	17.50%	15%	4.00	N/A	N/A	N/A
Mebrahatom et al*.* (28)	R. cross-sectional	AA	142	99.00	43.00	48.60%	20.40%	31%	14.00	12.00	1.00	1.00

*AA* Addis Ababa, *ACS* acute coronary syndrome, *N/A* not applicable, *STEMI* non-ST-elevation myocardial, *NSTEMI* ST-elevation myocardial infarction, *R* retrospective, *P* prospective, *UA* unstable angina

### Magnitudes of risk factors of ACS in Ethiopia

**Fig. 3:**
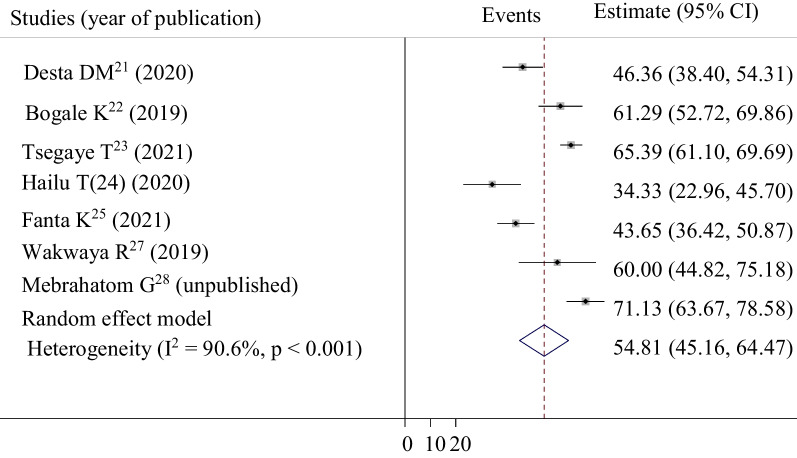
Forest plot of the pooled estimate of percentage hypertension among acute coronary syndrome patients in selected studies

**Fig. 4:**
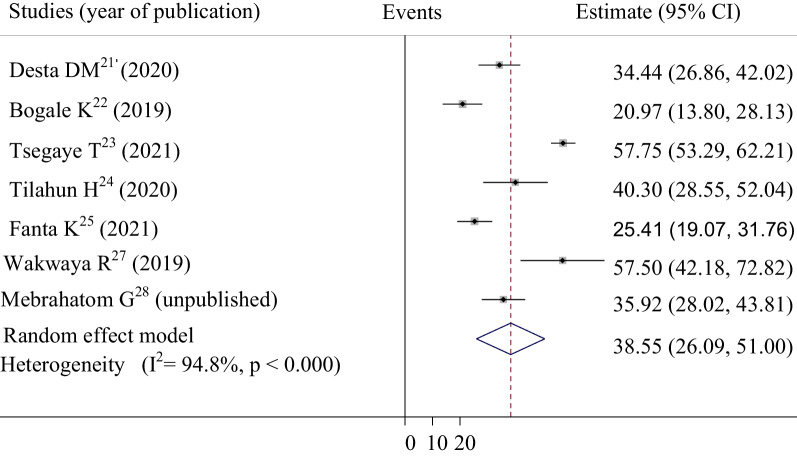
Forest plot of the pooled estimate of percentage diabetes mellitus among acute coronary syndrome patients in selected studies

**Fig. 5:**
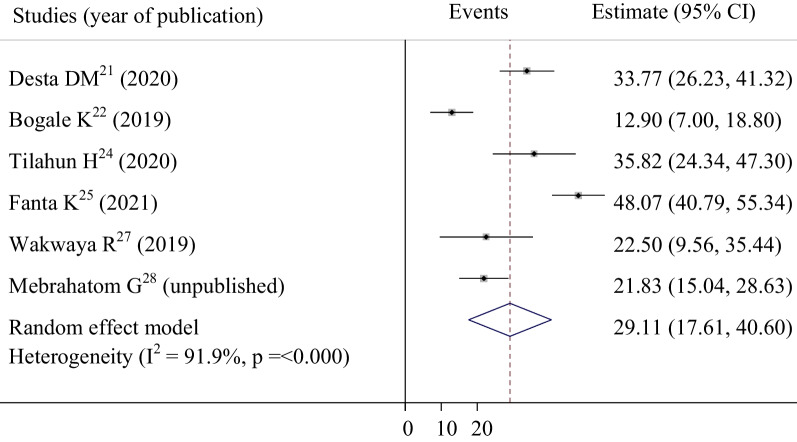
Forest plot of the pooled estimate of percentage dyslipidemia among acute coronary syndrome patients in selected studies

Patterns of risk factors among ACS patients in selected studies are shown in Table 2 and Additional file 2. Hypertension, diabetes mellitus, and dyslipidemia were the three most prevalent risk factors of ACS in Ethiopia, respectively. A total of 7 studies with 1176 study participants were included to assess the pooled magnitude of hypertension, and diabetes mellitus among ACS patients. Accordingly, our pooled analysis showed that more than one-half (54.814%, 95% CI: 45.158–64.470) of ACS patients had hypertension (Fig. 3). The pooled prevalence of diabetes mellitus in this systematic review and meta-analysis, we found that was 38.549% (95% CI: 26.095–51.004) (Fig. 4). To determine the pattern of dyslipidemia among ACS patients, a total of 2018 participants were included. Consequently, the result showed that nearly one-third of ACS patients (29.108%, 95% CI: 17.612–40.604) had dyslipidemia (Fig. 5).

**Table 2:** Pooled prevalence of risk factors among ACS patients in Ethiopia

S. no	Sample size	Risk factors	Pooled prevalence	95% confidence interval	References
1	1176	Hypertension	54.814%	45.158–64.470	[21–25, 27, 28]
2	1176	Diabetes mellitus	38.549	26.095–51.004	[21–25, 27, 28]
3	2018	Dyslipidemia	29.108	17.612–40.604	[21, 22, 24, 25, 27, 28]
4	191	Obesity	15.663	5.340–36.662	[21, 27]
5	746	Heart failure	19.599	4.941–44.138	[21–23]
6	1176	Smoking	19.985	10.475–29.495	[21–25, 27, 28]
7	686	Past history of MI	21.567	15.668–27.466	[21, 22, 24, 25, 27, 28]
8	24	Exertional angina pectoris	8.354	4.306–12.402	[21, 22]
9	372	Family history of CAD	12.319	0.815–23.823	[21, 25, 27]

*ACS* acute coronary syndrome, *CAD* coronary artery diseases, *MI* myocardial infarction

### In-hospital management of ACS based on types of ACS in Ethiopia

To determine the medication use pattern, we conducted a subgroup analysis based on diagnostic types of ACS. Anti-platelets, aspirin [56.903% (95% CI: 38.032–75.774)] and clopidogrel [55.266% (95%CI: 35.946–74.58)] were most frequently used in patients with STEMI than NSTEMI and UA. Similarly, more than one-half of patients with STEMI received beta-blockers (BBs) and statins. Primary percutaneous coronary intervention (PCI) was done in 24.558% (95% CI: 3.495–45.621) of patients with STEMI. Patients with UA had most (17.908%) commonly used anti-pain including morphine or fentanyl as compared to patients with NSTEMI (12.009%). Calcium channel blockers were prescribed inpatient with STMEI and UA almost in similar proportion (Additional file 2, Table 3).

**Table 3:** Subgroup analysis of articles describing medications used based on types of ACS

Type of ACS	Management	Total sample size	Frequency of medication used	Pooled prevalence (95%CI)	References
STEMI	Aspirin	1069	522	56.903 (38.032–75.774)	[21–23, 25, 28]
	Clopidogrel	1069	502	55.266 (35.946–74.586)	[21–23, 25, 28]
	Heparin	1069	446	47.273 (34.865–59.680)	[21–23, 25, 28]
	BB	598	307	51.591 (41.320–61.862)	[21, 22, 25, 28]
	ACEIs/ARBs	1069	450	43.441 (35.609–51.272)	[21–23, 25, 28]
	Nitrates	589	135	22.823 (14.240–31.406)	[21, 22, 25, 28]
	Statins	1069	473	51.803 (33.990–69.617)	[21–23, 25, 28]
	CCBs	888	85	7.782 (− 0.473–16.038)	[21–23, 28]
	Anti-pains	1069	276	25.505 (17.549–33.460)	[21–23, 25, 28]
	PCI	456	206	24.558 (3.495–45.621)	[21, 22, 25]
NSTEMI	Aspirins	1069	266	23.130 (15.545–30.714)	[21–23, 25, 28]
	Clopidogrel	1069	244	21.098 (14.001–28.195)	[21–23, 25, 28]
	Heparin	1069	231	19.891 (14.158–25.624)	[21–23, 25, 28]
	BB	598	181	18.734 (10.020–27.448)	[21, 22, 25, 28]
	ACEIs/ARBs	1069	198	16.963 (10.290–23.635)	[21–23, 25, 28]
	Nitrates	589	47	9.890 (4.206–15.574)	[21, 22, 25, 28]
	Statins	1069	262	21.779 (14.446–29.113)	[21–23, 25, 28]
	Anti-pains	1069	171	12.009 (3.179–20.840)	[21–23, 25, 28]
UA	Aspirin	888	249	22.703 (8.816–36.590)	[21–23, 28]
	Clopidogrel	888	214	20.666 (5.646–35.686)	[21–23, 28]
	Heparin	888	202	16.282 (1.754–30.809)	[21–23, 28]
	BB	417	66	14.331 (5.416–23.246)	[21, 22, 28]
	ACEIs/ARBs	888	197	15.365 (2.619–28.112)	[21–23, 28]
	Nitrates	417	28	5.960 (0.359–11.561)	[21, 22, 28]
	Statins	888	214	19.897 (7.262–32.532)	[21–23, 28]
	CCBs	888	81	6.302 (− 1.493–14.097)	[21–23, 28]
	Anti-pains	764	174	17.908 (2.402–33.414	[21, 23, 28]

*ACS* acute coronary syndrome, *ACEIs/ARBs* angiotensin-converting enzyme inhibitors/angiotensin receptor blockers, *BBs* beta-blockers, *CCBs* calcium channel blockers, *CI* confident interval, *PCI* percutaneous coronary intervention, *STEMI* non-ST-elevation myocardial, *NSTEMI* ST-elevation myocardial infarction, *UA* unstable angina

### In-hospital mortality of patients with ACS in Ethiopia

**Fig. 6:**
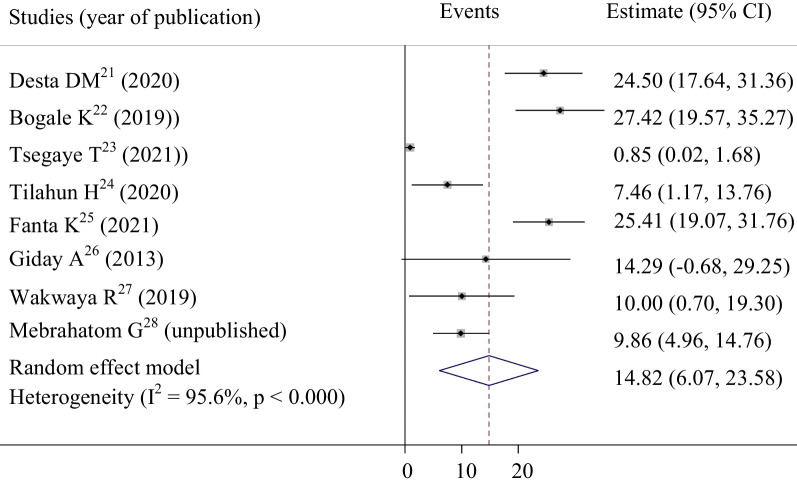
Overall all in-hospital mortality among acute coronary syndrome patients in Ethiopia

In this meta-analysis, we used a total of 1197 ACS patients to determine the overall mortality rate of ACS in Ethiopia. Accordingly, the pooled magnitude of in-hospital mortality of ACS was 14.82% (95% CI: (6.06–23.57) (Fig. 6). Results observed from subgroup analysis showed that in-hospital mortality was greatly varied across different types of ACS. The result indicated that patients with STEMI the highest magnitude of in-hospital mortality was reported in which was 16.156% (95% CI: 9.729–22.503) (Table 4).

**Table 4:** Subgroup analysis of studies included on in-hospital mortality by types of ACS

Subgroup	Included studies	Sample size	In-hospital mortality (95% CI)	References	
STEMI	5	619	16.116 (9.729–22.503)	[21, 22, 25, 26, 28]	
NSTEMI	5	619	2.867 (0.596–5.138)	[21, 22, 25, 26, 28]	
UA	3	417	0.969 (0.030–1.908)	[21, 22, 28]	

## Discussion

Currently, ACS is the leading cause of morbidity, mortality, and, high healthcare cost expenditure in resource-limited countries including Ethiopia. Identification of the risk factors of ACS may be important for clinicians and clinical pharmacists to set effective education programs and appropriate initial treatment. This review summarized magnitudes of risk factors, treatment approach, and prevalence of in-hospital mortality among patients with ACS in Ethiopia. Accordingly, the results of this study indicated that hypertension was the most commonly noted risk factor (54.814%) for ACS among included studies. The result was in line with studies conducted in Canada (59.7%), Sweden (58%), Greece (58.8%), and Kenya (55.56) [30–33].

In contrast, the proportion of hypertension observed in this systematic review and meta-analysis was lower than in studies done in Slovakia (83.5%) and Albania (90.6%) [11, 14]. This variation might be due to lack of diagnostic modalities and proficiency in our setting. On the other hand, a study conducted in Mexico showed that cigarette smoking (69.1%), hypertension (57.8%), and dyslipidemia (47.5%) were the three most prevalent risk factors among patients with ACS [34]. Similarly, a study done in South Africa among Asian Indian patients showed that 82% of ACS patients had visceral obesity as the number one risk factor and 60% of participants were cigarette smokers (60%) [35]. Our findings indicated that the prevalence of smoking and obesity were 19.985%, and 15.663%, respectively. Difference in genetic makeup and lifestyle condition might be the possible cause of variations. Diabetes mellitus and dyslipidemia were also the major risk factors for ACS, accounted 38.549%, and 29.108% of risk factors, respectively. A study conducted in Iran showed that the prevalence of hyperlipidemia was 45.9%, and diabetes mellitus was 31.1% [36]. Similarly a report from Japan, Prevention of Atherothrombotic Incidents Following Ischemic Coronary Attack (PACIFIC) Registry, indicated that dyslipidemia and diabetes mellitus were the main risk factors of ACS, 67.2% and 35.0%, respectively [37]. In addition, many other worldwide studies explained dyslipidemia and diabetes mellitus as common risk factors [15, 31, 32, 34]. These might be because dyslipidemia and diabetes mellitus have co-existed risk profiles and associated structural and functional changes in the cardiovascular system with devastating clinical complications [8, 13].

Pharmacological and invasive procedural therapy including percutaneous coronary intervention (PCI) or fibrinolysis is very essential to decrease in-hospital mortality [15, 30]. The finding of our study revealed that anti-platelets including aspirin and clopidogrel were most frequently used in patients with STEMI than the other two types of ACS, 56.903% and 55.266%, respectively. These were very lower as compared to finding from the Saudi project for assessment of coronary events (SPACE) registry, 98.4%, and 80.1%, respectively [38]. The use of aspirin and clopidogrel were also lower than in studies conducted in Iran (99.4% vs. 98.1%), Brazil (97.6% vs. 88.3%), and Spain (98.0% vs. 97.8%) [36, 39, 40]. This may give insight into the presence of gaps in the use of guideline-directed in-hospital management of ACS in Ethiopia which might be interesting for the future researcher. Different guidelines including the 2020 European Society of Cardiology Clinical Practice Guidelines recommended antiplatelet for all patients with ACS without contraindications, regardless of the type of ACS or the management strategy [41]. Antiplatelet are the cornerstone of ACS management because they significantly reduce the composite outcome of cardiovascular death, myocardial infarction, and stroke. It also considerably lowers the risk of recurrent ischemic events, including stent thrombosis [31, 42].

The use of beta-blockers in STEMI was 51.591% which was lower than studies from Iran (90.9%) and Brazil (80.9%) [36, 39]. The same is true for the use of statins, which was 51.803%. This was also lower than findings from India (84.3%), and Japan (80. 4%) [7, 37]. This variation might be due to differences in access to care, underlying risk factors, costs, and inconstant availability of drugs in Ethiopia. PCI is the most effective reperfusion therapy for symptomatic ACS patients admitted within 90 h [16]. However, only 24.558% of patients with STEMI received PCI therapy. The result was much lower as compared to studies conducted in Poland (55.5%) and Saudi Arabia (42.6%) [9, 38]. Importantly; this result was scored in the absence of using a fibrinolysis agent in Ethiopia. This might be because of problems with PCI service accessibility, limited interventional cardiologists, and delayed admission time to the hospital (range from 4 to 7 days) [22, 28]. Now a day, there are only two PCI-centered public hospitals in Ethiopia [43].

In this review, the overall in-hospital mortality due to ACS was 14.82%. The finding was in line with studies conducted in Pakistan (12.2%), Kenya (17%), and sub-Saharan African countries (10%) [44–46]. In contrast, in-hospital mortality found in this study was higher as compared to studies conducted in India (3.9%), China (7.66%), Netherland (3.7%), and Iraq (7.7%) [7, 47–49]. The possible causes of variation might be because of delayed initiation of diagnostic and treatment options in our setting. In addition, it might be due to the better availability of reperfusion therapy (PCI, coronary artery bypass graft, and thrombolytic therapy) in those listed countries. Patients with STEMI had higher (16.15%) rate of in-hospital mortality. In contrast, in a study in a central European country, results of the CZECH-2 registry, the rate of mortality was higher in patients with NSTEMI (8.4% for NSTEMI patients, 7.3% for STEMI patients) [50]. The variation might be due to the majority of patients in our study having STEMI (59.367%). The other possible reason for the higher mortality rate in STEMI subtypes of ACS might be the presence of complete blockage of the coronary artery as compared to partial occlusion in NSTEMI and UA.

### Limitations of the study

This systematic review and meta-analysis are not free from limitations. The first one is due to the presence of a weak database management system in our country, there may be data not accessible by our search strategy and therefore important measurements, particularly on mortality in each type of ACS might have been missed. In addition, most of the studies done in Ethiopia did not consider the specific name of the medication, dose, and duration of treatment. Due to these, we did not systematically capture data on these key parameters which might have been useful for creating better predictions on in-hospital management of ACS. Moreover, as the study relies on the articles conducted in Ethiopia with variability in study methodologies, the pooled prevalence estimates and conclusions may not be generalized to the rest of the world.

Time to present at a health institution after the onset of signs and symptoms is also a matter which is not addressed by this review. Finally, data were obtained from articles conducted with different methodologies and geographical regions; these may have an impact on the variation in magnitude of risk factors and practice in hospital management. Finally, both published and unpublished articles were included with different methodologies and geographical regions; these may have an impact on the variation in magnitude of risk factors and practice in hospital management.

However, this review provides useful information about the patterns of risk factors that contributed to the development of ACS. Besides, the review provides insight into the burden of ACS-associated mortality at the national level to help policymakers to design cost-effective plans and treatment strategies to combat the ACS burden and improve treatment outcomes.

## Conclusion

ACS is a major public health problem in Ethiopia with diverse clinical risk factors. Hypertension was the leading risk factor noted for ACS followed by diabetes mellitus and dyslipidemia. The use of guideline-directed in-hospital management was not comparable to that of registries from different countries in the world. Even though few patients were managed by PCI, we may conclude that the reperfusion therapy rate was almost zero in the absence of a fibrinolysis agent. The rate of in-hospital mortality among ACS patients is still high and the risk of death was higher in patients with STEMI. Initiation of treatment in clinical practice must take into account the heterogeneity of each patient’s risk factor. Health-related policymakers should work on the wide accessibility for the advanced therapies of PCI and thrombolytic following the patients’ socioeconomic status, which ultimately may help improve favorable patients’ in-hospital outcomes.

### Supplementary Information

**Additional file 1:** PRISMA checklist summarizing the quality of articles included.

**Additional file 2:** Magnitudes of risk factors among Acute coronary syndrome in Ethiopia.

## Data Availability

Additional data are supplied as additional material.
