# Supplementary material for: Acute coronary syndrome and its treatment outcomes in Ethiopia: a systematic review and meta-analysis
Source: J Pharm Policy Pract. 2023 Aug 7;16:98. doi: 10.1186/s40545-023-00603-7 (PMC10408155; doi:10.1186/s40545-023-00603-7)
Supplement: Supplementary file 1 — Additional file 1: PRISMA checklist summarizing the quality of articles included. [file 40545_2023_603_MOESM1_ESM.doc]

| **Section/topic** | **#** | **Checklist item** | **Reported on page #** |
| --- | --- | --- | --- |
| **TITLE** | | |  |
| Title | 1 | **The Magnitude of Risk Factors, Management and in Hospital Mortality of Acute coronary syndrome in Ethiopia: a systematic review and Meta-analysis.** | 1 |
| **ABSTRACT** | | |  |
| Structured summary | 2 | **Background:** Acute coronary syndrome (ACS) is the leading cause of death in developing countries including Ethiopia. Though several studies were conducted in Ethiopia, it is difficult to estimate the overall patterns of risk factors and burden of in-hospital mortality because of the lack of a nationwide study.  **Objective**: to assess the magnitude of risk factors, management, and in-hospital mortality of ACS in Ethiopia.  **Methods:** Electronic searching using PubMed, Science Direct, EMBASE, Scopus, Hinari, and Google Scholar for free articles were searched. Each of the original studies was identified by Boolean search techniques and the data were extracted using Preferred Reporting Items for Systematic Reviews and Meta-Analysis requirements. Data were pooled and a random effect meta-analysis model was applied to estimate the pooled prevalence risk factors and in-hospital mortality of ACS. Subgroup analysis was considered to examine how medications use and in hospital, mortality varies across different studies based on types of ACS.  **Results:** Most of the patients had ST-segment elevation myocardial infarction (STEMI) (59.367 %). Hypertension (54.814 %) was the most common risk factor of ACS followed by diabetes mellitus (38.549 %). Aspirin and clopidogrel were most frequently used in the patient with STEMI ACS, 56.903 %, and 55.266% respectively. The primary percutaneous coronary intervention was done in 24.558% of patients. The pooled magnitude of in-hospital mortality of ACS was 14.82 %, and it was higher in patients with STEMI (16.116 %)  **Conclusion**: ACS is a major public health problem in Ethiopia with diverse clinical risk factors. The use of guideline-directed in-hospital management was not comparable to other registries in different countries. The rate of in-hospital mortality is still high and it was higher in patients with STEMI. Initiation of treatment must consider the heterogeneity of each patient’s risk factor and reperfusion therapy should be implemented in our setting. | 2 |
| **INTRODUCTION** | | |  |
| Rationale | 3 | Acute Coronary Syndrome (ACS) is a coronary artery disease caused by narrowing or blockage of the coronary artery lumen, resulting in myocardial ischemia or infarction due to insufficient coronary blood perfusion.^1-2^ ACS, including unstable angina (UA), non-ST-elevation myocardial infarction (NSTEMI), and ST-elevation myocardial infarction (STEMI) is the main cause of cardiovascular death and disability in the world.^3-4^ It is associated with an average of 7.4 million deaths worldwide, 21% -22% of all deaths in Europe, and 6%–10% death in Sub-Saharan Africa.^5-6^  It is an emergency, life-threatening condition, and financial catastrophe due to high out-of-pocket expenditures for in-hospital care.^7-8^ Even though, there is advancement in diagnosis and management of ACS, still it is a leading cause of death around the globe including in Ethiopia.^9-10^ A study conducted at Addis Cardiac Hospital, Ethiopia showed that among 300 cardiac patients, 162(53.7%) of them had ACS.^11^ Moreover lower-income countries are facing a double burden of disease because of the higher prevalence of both communicable and non-communicable diseases particularly cardiovascular diseases.^10^ These result in frequent hospitalizations, high mortality rate, stretching the already limited resources, and associated with worse treatment outcomes.^9,12^  Even though ACS is a common health problem with devastating consequences, its burden and risk factors are different according to geographical variation.^13-14^ More than 90% of risk factors are potentially modifiable risk factors using pharmacological and non-pharmacological methods. However, it is difficult to achieve these outcomes in resource-limited countries because of insufficient laboratory setup, lack of reperfusion therapy, cost of medications, and discontinuity of care.^11, 15, 16^ Though several studies were conducted to describe the magnitude of risk factors, management and in-hospital mortality of acute coronary syndrome in Ethiopia, there is no a nationwide study on this area; which is an important research gap. Meta-analysis is a key to improving the accuracy of estimates through the use of more data sets.. | 3 |
| Objectives | 4 | This systematic review and meta-analysis was aimed to determine the overall magnitude of risk factors, management, and in-hospital mortality of acute coronary syndrome in Ethiopia. | 3 |
| **METHODS** | | |  |
| Protocol and registration | 5 | A review protocol did not exist | - |
| Eligibility criteria | 6 | All available studies and data were incorporated based on the following predefined eligibility criteria. All available studies and data were incorporated based on the following predefined eligibility criteria  **Inclusion criteria**   - **Study setting and period**: all studies conducted in Ethiopia from 2000 to November 30, 2021 - **Study design**: all facility-based observational studies - **Study population**: age ≥18 years old - **Article types**: The published and unpublished studies reporting the risk factors of ACS, management, and/or in-hospital mortality - **Language:** All searches were limited to articles written in the English language.   **Exclusion criteria**  We excluded reviews and systematic review articles, case reports, and case series. In addition articles available only in abstract form were excluded because it was difficult to evaluate their quality and extract all necessary information. If multiple publications the same studies with the same authors, only the most recent or most complete publication for each data set for a specific outcome was selected | 4-5 |
| Information sources | 7 | A comprehensive search for studies was done USING the following electronic databases from October 15 to November 30, 2021. PubMed, Science Direct, EMBASE, Cochrane Database, Sci-Hub, Scopus, Africa journal of online library, Hinari, and Google Scholar for free articles were searched. In addition, Addis Ababa, Jimma, and Gondar Universities institutional repositories were considered to address unpublished articles in Ethiopia. | 4 |
| Search | 8 | First, articles were searched by examining the full titles ("Magnitude of Risk Factors, Management and in Hospital Mortality of Acute coronary syndrome in Ethiopia”) and then keywords (magnitude, risk factors, treatment, mortality, acute coronary syndrome, coronary heart disease, Ethiopia). These different keywords were used individually and in combination using Boolean operators "OR, AND or NOT" as well as medical subject heading [MeSH] terms). In addition, searching reference lists of all the included studies (snowball technique) was done to retrieve other studies that are not addressed by our searching stratagem | 4 |
| Study selection | 9 | Essential data were extracted from eligible studies by using Microsoft Excel spreadsheet format. To ensure data quality and methodological validity, two authors (BK and MG) were retrieved the data independently. Data were extracted using the PRISMA standard data extraction format. Data extraction quality was accompanied in according to the Critical Appraisal Checklist recommended by the Joanna Briggs Institute (JBI).^18^ The JBI checklists was composed of ten questions, the scores ranged from zero to ten. The studies which obtained more than 60% were considered as good quality studies. None of them had poor quality status and all of them were included in the present inquiry | 5 |
| Data collection process | 10 | All articles included in the final analysis were reviewed by two authors independently using Microsoft Excel spreadsheet format. The following information were considered during data extraction: The last name of the first author and year of publication, the region of the study conducted, study design and period, total sample size, sex, types of ACS, risk factors of ACS, the medication used, overall in-hospital mortality and mortality in each type of ACS. Any discrepancies in the data extraction process were solved through discussion involving all authors. | 5 |
| Data items | 11 | Magnitudes of risk factors, management and mortality of ACS | 5 |
| Risk of bias in individual studies | 12 | To minimize selection bias, all possible relevant articles were evaluated critically by two reviwers independently and those that meet the inclusion criteria were selected. The JBI checklist was composed of ten questions, the scores ranged from zero to ten. The studies which obtained more than 60% were considered as good quality studies | 6 |
| Summary measures | 13 | Data were pooled and a random effect meta-analysis model was applied to estimate the pooled prevalence risk factors and in-hospital mortality of ACS, and the result was presented using forest plot and odds ratio(OR) with 95% confident interval(CI) | 6 |
| Synthesis of results | 14 | Heterogeneity between studies was assessed by computing chi-square (I^2^) test statistics. The I^2^ values of 0, 25, 50, and 75% were considered as no, low, moderate, and high heterogeneities, respectively. | 6 |

Page 1 of 2

| **Section/topic** | **#** | **Checklist item** | **Reported on page #** |
| --- | --- | --- | --- |
| Risk of bias across studies | 15 | Begg’s funnel plot and Egger's regression were used to check publication bias and a p-value less than <0.05 was considered as statically significant | 6 |
| Additional analyses | 16 | Subgroup analysis was considered to examine how medications used and in hospital, mortality varies across different studies based on types of ACS. | - |
| **RESULTS** | | |  |
| Study selection | 17 | We identified a total of, 271 published and 4 gray kinds of literature through database searches. After articles were removed by duplications, title, and reading the abstract, 24 studies were assessed for eligibility criteria. Consequently, 16 articles were excluded due to different reasons because they were irrelevant. Finally, a total of 8 studies met the inclusion criteria and were included in the final analysis (Figure 1). | 7 |
| Study characteristics | 18 | The articles were published between 2013 and 2021; however, there is one unpublished article that was obtained from the Addis Ababa University repository. Most of the studies (6/8, 75%) were cross-sectional studies, others were longitudinal or retrospective follow-up studies. More than one-half of the studies were conducted in Addis Ababa city (n = 6) others were conducted in the Oromia region (n = 1), and Tigray region (n = 1). From a total of 1197 study participants, 813(67.92%) of them were females (Additional file 1). With regarding to diagnostic types of ACS, majority of patients had STEMI (59.367%, 95% CI: 44.558-74.176) followed by NSTEMI (23.365%, 95% CI: 16.559-30.171) and then UA (19.899%, 8.092- 31.706) (Table 1). | 7 |
| Risk of bias within studies | 19 | The result of both Begg’s and Egger's tests showed that there was considerable publication bias at <0.001 p-values and <0.006 respectively and assessment of publication bias using funnel plot in (Figure 2) | - |
| Results of individual studies | 20 | Patterns of risk factors among ACS patients in selected studies are shown in (Table 2 and additional file 1). Hypertension, diabetes mellitus, and dyslipidemia were the three most prevalent risk factors of ACS in Ethiopia, respectively. A total of 7 studies with 1176 study participants were included to assess the pooled magnitude of hypertension, and diabetes mellitus among ACS patients. Accordingly, our pooled analysis showed that more than one-half (54.814%, 95% CI: 45.158- 64.470) of ACS patients had hypertension (Figure 3). In this systematic review and meta-analysis, we found that the pooled prevalence of diabetes mellitus was 38.549 % (95% CI: 26.095-51.004) (Figure 4). To determine the pattern of dyslipidemia among ACS patients, a total of 2018 participants were included. Consequently, the result showed that nearly one-third of ACS patients (29.108%, 95% CI: 17.612-40.604) had dyslipidemia (Figure 5). | 7-10 |
|  |  |  | 07-14 |
| Synthesis of results | 21 | In this meta-analysis, we used a total of 1197 ACS patients to determine the overall mortality rate of ACS in Ethiopia. Accordingly, the pooled magnitude of in-hospital mortality of ACS was 14.82 %( 95% CI: (6.06-23.57) (Figure 6). Results observed from Subgroup analysis showed that in-hospital mortality was greatly varied across different types of ACS. The result indicated that the highest magnitude of in-hospital mortality was reported in patients with STEMI which was 16.116 % (95% CI: 9.729-22.503) (Table 4). | - |
| Risk of bias across studies | 22 | Begg’s funnel plot and Egger's regression were used to check publication bias and a p-value less than <0.05 was considered as statically significant | - |
| Additional analysis | 23 | To determine the medication use pattern, we conducted a subgroup analysis based on diagnostic types of ACS. Anti-platelets, aspirin (56.903 %( 95% CI: 38.032- 75.774)) and clopidogrel (55.266% (95%CI: 35.946 - 74.58)) were most frequently used in patients with STEMI than NSTEMI and UA. Similarly, more than one-half of patients with STEMI received beta-blockers (BBs) and statins. Primary percutaneous coronary intervention (PCI) was done in 24.558% (95% CI: 3.495 -45.621) of patients with STEMI. The use of anti-pain including morphine or fentanyl was higher among patients with UA (17.908%) as compared to patients with NSTEMI (12.009%). Calcium channel blockers were prescribed inpatient with STMEI and UA almost in similar proportion (Additional file 2, Table 3). | - |
| **DISCUSSION** | | |  |
| Summary of evidence | 24 | The results of this study indicated that hypertension was the most commonly noted risk factor (54.814%) for ACS among included studies. The result was in line with studies conducted in Canada (59.7%), Sweden (58%), Greece (58.8 %), and Kenya (55.56).^29-32^  In contrast, the proportion of hypertension observed in this systematic review and meta-analysis was lower than studies done in Slovakia (83.5%) and Albania (90.6%).^11, 14^ This variation might be due to lack of diagnostic modalities and proficiency in our setting. On the other hand, a study conducted in Mexico showed that cigarette smoking was the leading cause of ACS (69.1%).^33^ Similarly, a study in South African among Asian Indian patients showed that 82% of ACS patients had visceral obesity as the number one risk factor and 60% of participants were cigarette smokers (60%).^34^  Our findings indicated that the prevalence of smoking and obesity were 19.985%, and 15.663%, respectively. Difference in genetic makeup and lifestyle condition might be the possible cause of variations. Diabetes mellitus and dyslipidemia were also the major risk factors for ACS, accounted 38.549 %, and 29.108% of risk factors, respectively. A study conducted in Iran showed that the prevalence of hyperlipidemia was 45.9%, and diabetes mellitus was 31.1%.^35^ Similarly a report from Japan, Prevention of Atherothrombotic Incidents Following Ischemic Coronary Attack (PACIFIC) Registry, indicated that dyslipidemia and diabetes mellitus were the main risk factors of ACS, 67.2% and 35.0 % respectively.^36^ In addition, many other worldwide studies explained dyslipidemia and diabetes mellitus as common risk factors.^15,30,31,33^ These might be because dyslipidemia and diabetes mellitus have co-existed risk profiles and associated structural and functional changes of the cardiovascular system with devastating clinical complications.^8,13^  Pharmacological and invasive procedural therapy including percutaneous coronary intervention (PCI) or fibrinolysis is very essential to decrease in-hospital mortality.^15,29^ The finding of our study revealed that Anti-platelets including aspirin and clopidogrel were most frequently used in patients with STEMI than the other two types of ACS, 56.903 %and 55.266% respectively. These were very lower as compared to finding from the Saudi project for assessment of coronary events (SPACE) registry, 98.4%, and 80.1% respectively.^37^ The use of Aspirin and clopidogrel were also lower than studies conducted in Iran (99.4% v.98.1%), Brazil (97.6% VS.88.3%), and Spain (98.0% vs., 97.8 %).^35,38,39^ This may give insight into the presence of gabs in the use of guideline-directed in-hospital management of ACS in Ethiopia which might be interesting are for the future researcher. Different guidelines including the 2020 European Society of Cardiology Clinical Practice Guidelines recommended antiplatelet for all patients with ACS without contraindications, regardless of the type of ACS or the management strategy.^40^ Antiplatelet are the cornerstone of ACS management because they significantly reduce the composite outcome of cardiovascular death, myocardial infarction, and stroke. It also considerably lowers the risk of recurrent ischemic events, including stent thrombosis.^30, 41^  The use of beta-blockers in STEMI was 51.591% which was lower than studies from Iran (90.9%) and Brazil (80.9%).^35, 38 The^ same is true for the use of statins which was 51.803%. This was also lower than findings from Indian (84.3%), and Japan (80. 4%).^7,36^  This variation might be due to differences in access to care, underlying risk factors, costs, and inconstant availability of drugs in Ethiopia. PCI is the most effective reperfusion therapy for symptomatic ACS patients admitted within 90 hours.^16^  However, only 24.558% of patients with STEMI were received PCI therapy. The result was much lower as compared to studies conducted in Poland (55.5%) and Saudi Arabia (42.6%).^9, 37^ Importantly; this result was scored in the absence of using a fibrinolysis agent in Ethiopia. This might be because of problems with PCI service accessibility, limited interventional cardiologists, and delayed admission time to the hospital (range from 4- 7 days).^21,27^  Now a day, there are only two PCI-centered in public hospitals of Ethiopia.^42^  In this review, the overall in-hospital mortality due to ACS was 14.82%. The finding was in line with studies conducted in Pakistan (12.2%), Kenya (17%), and Sub-Saharan African countries (10%).^43-45^ In contrast, in-hospital mortality found in this study was higher as compared to studies conducted in Indian (3.9%), China (7.66%), Netherland (3.7%), and Iraqi (7.7%).^7,46,47,48^ The possible causes of variation might be because of delayed initiation of diagnostic and treatment options in our setting. In addition, it might be due to the better availability of reperfusion therapy (PCI, coronary artery bypass graft, and thrombolytic therapy) in those listed countries. The rate of in-hospital mortality was higher among patients with STEMI which was 16.116%. In contrast, in a study in a central European country,Results of the CZECH-2 registry, the rate of mortality was higher in patients with NSTEMI (8.4% for NSTEMI patients, 7.3% for STEMI patients).^49^ The variation might be due to the majority of patients in our study having STEMI (59.367%). The other possible reason for the higher mortality rate in STEMI subtypes of ACS might be due to the presence of complete blockage of the coronary artery as compared to partial occlusion in NSTEMI and UA. | 12-14 |
| Limitations | 25 | This systematic review and meta-analysis are not free from limitations. The first one is due to the presence of a weak database management system in our country, there may be data not accessible by our search stratagem and therefore important measurement, particularly on mortality of in each type of ACS might have been missed. In addition, most of the studies done in Ethiopia did not consider the specific name of the medication, dose, and duration of treatment. Due to these, we did not systematically capture data on these key parameters which might have been useful for creating better predictions on in-hospital management of ACS. Time to present at health institution after the onset of signs and symptoms is also a matter which is not addressed by this review. Finally, data were obtained from articles conducted with different methodologies and geographical regions; these may have an impact on the variation in magnitude of risk factors and practice in hospital management. However, this review provides useful information about the patterns of risk factors that contributed to the development of ACS. Besides, the review provides insight into the burden of ACS associated mortality at the national level to help policymakers to design cost-effective plans and treatment strategies to combat ACS burden and improve treatment outcomes. | 14 |
| Conclusions | 26 | ACS is a major public health problem in Ethiopia with diverse clinical risk factors. Hypertension was the most common risk factor noted for ACS followed by diabetes mellitus and dyslipidemia. The use of guideline-directed in-hospital management was not comparable to that of registries from different countries in the world. Even though few patients were managed by PCI, we may conclude that the reperfusion therapy rate was almost zero in the absence of a fibrinolysis agent. The rate of in-hospital mortality among ACS patients is still high and the risk of death was higher in patients with STEMI. Initiation of treatment in clinical practice must take into account the heterogeneity of each patient risk factor. Health-related policymakers should work on the wide accessibility for the advanced therapies of PCI and thrombolytic following the patients' socioeconomic status, which ultimately may help improve favorable patients ‘in-hospital outcomes. | 14-15 |
| **FUNDING** | | |  |
| Funding | 27 | N/A | - |

N/A= Not applicable
